# Tax Talk: An Exploration of Online Discussions Among Taxpayers

**DOI:** 10.1007/s10551-016-3032-y

**Published:** 2016-02-19

**Authors:** Diana Onu, Lynne Oats

**Affiliations:** Tax Administration Research Centre, Business School, University of Exeter, Streatham Court, Rennes Drive, Exeter, EX4 4ST UK

**Keywords:** Tax behaviour, Tax compliance, Tax evasion, Online discussion, Thematic analysis

## Abstract

We present an analysis of over 400 comments about complying with tax obligations extracted from online discussion forums for freelancers. While the topics investigated by much of the literature on taxpayer behaviour are theory driven, we aimed to explore the universe of online discussions about tax in order to extract those topics that are most relevant to taxpayers. The forum discussions were subjected to a qualitative thematic analysis, and we present a model of the ‘universe’ of tax as reflected in taxpayer discussions. The model comprises several main actors (tax laws, tax authority, tax practitioners, and the taxpayer’s social network) and describes the multiple ways in which they relate to taxpayers’ behaviour. We also conduct a more focused analysis to show that the majority of taxpayers seem unconcerned with many of the variables that have been the focus of tax behaviour research (e.g. audits, penalties, etc.), and that most people are motivated to be compliant and are more concerned with *how* to comply than *whether* to comply. Moreover, we discuss how these ‘real-world’ tax discussions question common assumptions in the study of tax behaviour and how they inform our understanding of business ethics more generally.

## Introduction

*Whether you can observe a thing or not depends on the theory which you use. It is the theory which decides what can be observed.* (Albert Einstein).

While Einstein’s observation referred to quantum mechanics, it is none less true for any field of knowledge where systematic empirical testing of theory is central, including economics and social sciences. Theoretical frameworks focus researchers on specific variables, and in turn these researchers collect and analyse those data that are relevant to understanding their variables of interest. While such systematic testing of theory is essential, it also means that certain data are never observed because they are not the focus of current theories. In this paper, we explore one particular field of study and attempt to reveal such data that have escaped systematic observation due to the focus of current theories in the field: the study of taxpayer behaviour.

When the Australian billionaire and media tycoon Kerry Packer was asked about his tax affairs during a public inquiry, he said, “Now, of course, I am minimizing my tax, and if anybody in this country doesn’t minimize their tax they want their heads read”. While Kerry Packer’s position may seem controversial to some, it will seem natural to others; in the research environment, ‘head-reading’ efforts have attempted to establish the factors that explain this variation in how people make tax compliance decisions (for recent literature reviews, see Hashimzade et al. 2013; Kirchler 2007; Pickhardt and Prinz 2013).

Tax behaviour research has seen contributions from various disciplines, approaches, and theoretical positions. However, the field has been dominated by theoretical and laboratory-based research, research that is primarily driven by researchers’ assumptions (see Boll 2013; Oats 2012). The mainstream approach has been to test theoretically derived models of behaviour empirically, usually by eliciting people’s responses to a task (e.g. filling in a questionnaire, taking part in an experiment, etc.). Our approach in this paper is very different: we collect and analyse *naturally* *occurring data* on tax behaviour (online discussions) in a *qualitative* manner. We look at how people’s discussions confirm what we know about tax behaviour, and also if there are differences between how people talk about tax ‘in the real-world’ and current results and assumptions in the literature.

Some of our findings are in line with previous thinking about tax compliance behaviour; other findings indicate the need to revisit assumptions we make about phenomena we study. But, most of all, our interrogation of naturally occurring discussions reveals some blind spots in research on tax behaviour. We will dedicate the largest part of this paper to discussing findings of the analysis and their relationship with existing literature. Beforehand, we make a brief incursion into research on tax compliance to serve those readers less familiar with this field.

## Very Brief Overview of Tax Compliance Research

The first works to look at tax compliance as an individual psychological decision were published by Günter Schmölders from the 1950s onwards (see, for instance, Schmölders 1959). Although an economist himself, Schmölders (1959) advocated the need to use psychology to understand fiscal behaviour, and he was particularly interested in how people make compliance decisions based on their values or belonging to certain social groups (profession, ‘social class’, etc.). A number of later works answered his call, constructing models of tax compliance that would take into account the variety of psychological and social factors involved in compliance decisions (e.g. personal values, attitudes, social norms, peer effects, and other such factors) (see, for example, Lewis 1982; Weigel et al. 1987; for an overview, see Webley 1991). More recently, psychologists studying tax behaviour have looked in depth at several psychological factors related to compliance, such as social norms (e.g. Wenzel 2004), perceptions of tax authorities (e.g. Kirchler et al. 2008), stance towards authorities and government (Braithwaite 2009), and even the ‘mental accounting’ involved in dealing with one’s tax affairs (Adams and Webley 2001).

A different stream of tax compliance research was developed in economics in the early 1970s on the foundation of Gary Becker’s (1968) economics-of-crime model, a model that posits that criminal decisions can be viewed as rational choices: criminals weigh up the level of penalty and the probability of being caught, and make a rational decision following a cost-benefit analysis. This approach applied to tax evasion decisions has come to be known as the ‘classic model’ of tax compliance (Allingham and Sandmo 1972; Yitzhaki 1974). Many authors subsequently noted the failure of the classic model to predict realistic evasion levels (see Andreoni et al. 1998), and several attempts have been made to improve the model (for a review, see Hashimzade et al. 2013), for instance by considering the role of social norms in deterring tax evasion (Myles and Naylor 1996).

As with many branches of applied economics, the perceived failure of the ‘classic model’ based on taxpayers as rational actors was noted by behavioural economists (see Alm et al. 1995). Many behavioural studies instead framed evasion decisions in the context of public goods games (i.e. tax is seen as something that all benefit from; however, individuals may be tempted to evade paying their share and still get a share of everyone else’s contribution). Studies looked at how different variations of the public goods game alter people’s decision to evade contributing to the public good; such variations include, for example, participants having agency over penalty levels (Alm et al. 1999), the role of different tax rates for income brackets (Bosco and Mittone 1997), and others.

## Broad Assumptions in the Tax Compliance Literature

As pointed out elsewhere (Oats 2012), the vast majority of works looking at tax compliance are situated in a positivistic research paradigm, that is, they employ systematic data collection (e.g. survey, experiment) and/or mathematical analyses to validate research hypotheses. While this approach has clear benefits in providing rigorous answers, its drawback is that hypotheses rely heavily on researchers’ assumptions of what is important for tax behaviour. To give one example, as outlined above, Allingham and Sandmo (1972) posit that audits and penalties are essential to understanding compliance decisions. As such, researchers’ theoretical and empirical analyses will become geared towards refining our understanding of the role of audits and penalties. They, however, tell us nothing about the validity of our initial assumptions that audits and penalties are indeed important for the tax behaviour of most individuals. In a laboratory experiment, changing audit rates may produce an important effect on behaviour because we control the effect of all other factors, but in ‘the real-world’, outside the lab, audit rates may be one of the least important things considered by taxpayers. In the current study, we attempt to provide an exploration of what is important for taxpayers ‘in the real-world’, and compare the results of this exploration with current assumptions in the tax compliance literature. Before we describe our approach and method, we discuss below some *specific assumptions* that are common in tax compliance research.

1. *Regarding tax behaviour, people’s chief concern is deciding whether to comply or not* Most research has been geared towards understanding the tax compliance decision: to evade or not to evade (for reviews, see, for example, Andreoni et al. 1998; Kirchler 2007; Pickhardt and Prinz 2013). However, in reality, many taxpayers do not know and struggle to find out *how* to comply, rather than *whether* to comply, spending time on deciphering the complexity and abstractness of the tax code (Alm et al. 2010). Tax authorities estimate that a large section of people are willing to comply but do not know how to do so, a much larger proportion than people who would contemplate breaking the law (“SME Customer Segmentation”, 2010). Certainly, it is essential to study the compliance decision, but other processes may be just as important in taxpaying behaviour. In reality, the compliance decision may be irrelevant for a large swathe of taxpayers.
2. *Compliance is a binary variable* In order to operationalise the tax compliance decision as simply as possible, the vast majority of studies of tax compliance assume two distinct options for the individual: to evade or to be fully compliant. However, as some have pointed out, ‘real-world’ compliance is far from binary. The complexity of compliance is illustrated by the existence of different types of compliance (e.g. voluntary versus enforced compliance, see Braithwaite 2009; Kirchler and Wahl 2010), or by situations of taxpayers taking advantage of legal ‘grey areas’ to drastically minimise tax while still complying with the letter of the law (tax avoidance, Kirchler and Wahl 2010; creative compliance, McBarnet 2004). While tax evasion may be defined as evading taxes with intent (McBarnet 2004), it may be very difficult to determine whether misreporting on a tax return is due to evasion intent or error. In the attempt to simplify compliance to a binary decision for research purposes, it may be that such simplification bears little resemblance to the complexity of realistic tax compliance behaviour.
3. *The taxpayers and the tax authority are the main actors* The majority of studies have looked at the relationships between taxpayers and the tax authority, to the detriment of other ‘actors’ such as peer groups, accountants and legal advisors, etc. (for a review, see Pickhardt and Prinz 2013). Although it may seem that the actions of tax authorities are most important for tax compliance (i.e. by employing certain audit strategies, having a customer-focused approach, etc.), research has not focused in as much detail on the role of other ‘actors’.
4. *Taxpayers are concerned with audit rates and penalty levels* As several authors have noted (e.g. Andreoni et al. 1998; James and Alley 2002; Kirchler 2007), the field of tax compliance has been dominated by the economics-of-crime approach to tax evasion (Allingham and Sandmo 1972), that is, that taxpayers make evasion decisions following a cost-benefit analysis that takes into account the income loss if caught evading (penalty) and the probability of being caught (audited). In this paradigm, raising penalty levels and conducting more audits produces higher compliance levels; often called the deterrence approach (Alm and Torgler 2011), this paradigm stresses the importance of people’s assessment of penalty and audit levels for compliance decisions.
5. *Alternatively, taxpayers base their decisions on social norms* Many have noted the failure of the deterrence model to explain why some people would not evade taxes even if penalties and audits never existed (e.g. Wenzel 2004), as well as its failure to predict the high compliance levels observed in reality (Andreoni et al. 1998). As such, to explain why compliance levels are higher than expected, many researchers propose that social norms account for why so many people comply when the ‘rational’ choice would be to evade. Evasion is thought to be mitigated by strong norms against evasion (e.g. Bobek et al. 2007; Wenzel 2005), whether that is because people fear the negative consequences of reputation loss (Myles and Naylor 1996) or because strong norms signal that most other taxpayers are willing to contribute to the public goods (Frey and Torgler 2007).

## The Current Study

As outlined above, certain broad assumptions seem to underpin much of research on tax compliance. However, there is little exploration of the extent to which these assumptions are relevant for tax behaviour as a whole. As we briefly discussed, there is reason to doubt that some of these assumptions readily reflect the reality of paying tax for many individuals. To explore individuals’ reality in depth, qualitative methods of inquiry may prove more effective (for an overview of qualitative methods in tax research, see Oats 2012), particularly when the researcher is merely observing tax compliance processes without intervening (as opposed to, for instance, surveying taxpayers). Observation of naturally occurring behaviour has been used extensively in the social sciences (for an example of ethnographic observation of tax audits, see Boll 2013). In this study, we collect and analyse naturally occurring interactions, but in an online environment (what is sometimes termed ‘netnography’ in business and organisational studies, see Kozinets 2002; see also Garcia et al. 2009; Hine 2000). We present an analysis of discussions about tax on online forums for self-employed individuals; this analysis is not focused by particular hypotheses—it is an open exploration of the tax behaviour ‘universe’ as reflected in people’s discussions about tax.

We chose the virtual ethnographic approach given several advantages it presents. First, the approach allows us to collect data that have not been generated by a researcher and that reflect people’s naturally occurring concerns. Second, it provides some insight into taxpayers’ attitudes and opinions. People often discuss online with a high degree of self-disclosure, including sensitive topics, particularly when they address those they feel connected to (e.g. peers in the same profession), as opposed to disclosing to a researcher in a one-off survey or interview. Third, online communication is less hierarchical and may thus allow more freedom of expression than traditional research settings that place the researcher in an expert position (for overviews, see Hine 2013; Kozinets 2015).

## Method

### Data Collection

The dataset analysed consists of over 400 user comments about tax on discussions forums for self-employed professionals in the UK. The dataset comprises 144 conversations on these forums: a forum user asks a question or raises an issue related to tax which is followed by other users replying, debating, advising, sharing their own experience, etc. These online forums are not specialised tax forums, but general forums for individuals who work as freelancers and/or run micro-businesses, where individuals will discuss a wide range of issues (e.g. how to start and run a business, how to advertise products, etc.), including legal issues and taxation.

This particular data collection strategy was preferred for several reasons. First, we focused on general forums rather than those specialised on tax issues because we aimed to collect conversations relevant to people who have no expertise or keen interest in taxation. Second, we chose to focus on self-employed individuals, as they represent a relatively simple case for understanding tax compliance. This contrasts with taxation in small or even medium-sized businesses where decisions are more complex, often being distributed among directors, involving a larger number of taxes, etc. Mitigating factors such as reputation loss and the influence of norms take on a different hue when considering more formal organisations (for studies of organisational compliance see, for example, Edelman and Talesh 2011; Parker 2000). Third, we chose online discussion forums as opposed to other social media because discussions on these forums tend to be more elaborate than those usually posted on social networking sites, allowing more depth to the analysis.

We chose to focus on the UK rather than include more countries because narrowing the focus allowed a more comprehensive data collection strategy. Although we do not make any claims regarding how representative the comments collected are for all discussions about tax among UK self-employed professionals, we have endeavoured to select the most prominent forums for a range of occupations, and included all comments about tax on those forums. Most of the comments included in the analysis were collected on a general discussion forum for freelancers, but we also aimed to include specialised forums for particular professions (those included here are forums for artists, designers, construction industry professionals, nurses, doctors, beauticians and hairdressers, and IT contractors). The selection of these groups, however, is not intended to imply that behaviour in these groups is different from the wider population.

To make sure we include all potentially relevant discussions, we selected from the relevant forums all the discussions that contained the term ‘tax’, and subsequently eliminated from the dataset only those where ‘tax’ was used to refer to something other than taxation (e.g. people using ‘taxing’ to mean ‘demanding’). Data were collected in late 2013–early 2014, but some of the comments were posted as early as 2003, although most were much more recent. All the information collected is publicly available, and did not require the researcher to register as a forum user in order to access it.

### Thematic Analysis

To analyse the conversations, we employed thematic analysis (Braun and Clarke 2006). Considered the most widely used qualitative analysis method, thematic analysis provides a systematic approach to extract meaning from text data (Braun and Clarke 2006). We used qualitative analysis software (NVivo), and began by open-coding all the comments in the dataset (i.e. a process of assigning a label to each comment relevant to its content, for instance ‘tax authority’ or ‘fine’). This process allowed the creation of a set of categories that were further refined, that is, similar themes were merged, or large and heterogeneous themes were split into sub-themes. This iterative process of refining categories while ensuring they are true to the underlying data ultimately produced a number of themes that presents our interpretation of all the data collected.

As Braun and Clarke (2006) argue, because thematic analysis is a flexible method of analysis when compared to most other qualitative methods that have strict methodological and philosophical underpinnings, it is important for researchers to acknowledge their particular philosophy in analysing the data; our stance in this particular study is a realist one that assumes people’s talk is reflective of thoughts, attitudes, or motivations (Potter and Wetherell 1987; for a discussion of the different epistemologies in thematic analysis, see Braun and Clarke 2006).

## Results and Discussion

We begin by discussing the themes that emerged from our data analysis, and follow this presentation with a discussion of the broad assumptions in the tax compliance literature we identified in the introduction—whether and how these assumptions are reflected in naturally occurring discussions among taxpayers.

### Model and Findings

**Fig. 1:**
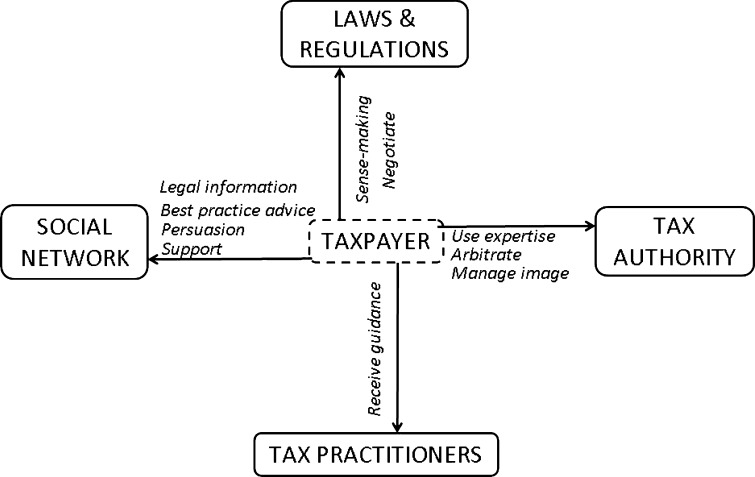
Theoretical model

The results provide an overview of the content of discussions analysed. Figure 1 presents the data categories and their relationships. Following presentation of the model, we discuss each theme in detail, providing quotations from the dataset to illustrate each of the themes (quotations have been paraphrased in order to maintain confidentiality).

The model presented in Fig. 1 serves to illustrate and organise the main themes in the dataset. To organise the themes, we identified the main ‘actors’ in these tax discussions, and then the sub-themes that reflect the relationship between the taxpayers and these ‘actors’. In the process of dealing with his or her tax affairs, the taxpayer deals with (1) the laws and regulations relating to tax compliance, (2) the tax authority, (3) tax practitioners, and (4) the wider social network of the taxpayer (for example, co-workers, members of the same professional group, family and friends, etc.). We focus below on how all these ‘actors’ are reflected in the comments we analysed and on describing the relationship between the taxpayer and each of the ‘actors’.

#### Laws and Regulations

The majority of comments (please see Table 1) included in the analysis referred to tax laws, rules, regulations, such as requirements to register with the tax authority when trading, deadlines for payment, types of taxes due, etc., as well as recent legal changes. One of the main concerns of taxpayers interacting on the online forums was to make sense of laws and regulations, receive uncomplicated explanations from other forum users and understand which rules apply to their particular situation. Others who are generally familiar with basic taxpaying rules may use online discussions to determine whether all general rules apply to their circumstances, or if they can make use of exemptions or even set up business structures that are tax-effective. Therefore, our dataset showed two main distinct ways for taxpayers to approach laws and regulations: (1) making sense of existing rules; or (2) negotiating the legal landscape by understanding if rules apply to their circumstance and how to follow rules that are in their favour. We briefly discuss these two types of approaching rules below.

**Table 1:** Occurrence of instances of themes in the dataset

Main themes and sub-themes	Occurrences in dataset
Laws and regulations	
Sense-making	345
Negotiation	233
Tax authority	
Use expertise	37
Arbitrate	31
Manage image	7
Tax practitioners	
Provide guidance	63
Social network	
Legal information	82
Best practice advice	77
Persuasion	17
Support	15
Other topics of interest	
Audits	14
Penalties	16
Norms of professional group	35

Each data point represents a contribution by a forum member. As such, each contribution may refer to several themes and be coded simultaneously as part of multiple categories

##### Sense-Making

Many of the online discussions analysed began with a user requesting advice in making sense of tax rules, as illustrated by the comments below:

1. I have ABSOLUTELY no clue when it comes to this sort of thing, I take it I have to register with [the tax authority] who will advise what I owe them. Can you guys give ANY advice on the matter or refer me to any books or articles.
2. I don’t know enough about exports from UK to US or Canada. I began reading some tax documents but my brain went into meltdown. Is there any import duty? I’d appreciate your advice?

Many comments stress that they find tax complicated, and the general information provided by the tax authority or by accountants, confusing.

1. The [tax authority] website is so complicated, I could not find any information relating to small items of equipment. [query regarding allowable expenses].
2. Taxes are one of the things that give us headaches.

As such, a large number of comments are dedicated to laying out the rules in simple terms, sometimes discussing and debating the accuracy of information presented.

1. yes you have to pay tax—you have to register as if you are selling work.
2. V.A.T is not needed… in fact, it’s likely you won’t need to be V.A.T. registered for the future either.
3. How much you have to pay the taxman is irrelevant, not declaring income is a crime, whether for millions or pennies. [responding to a debate regarding a minimal income for registration].

The sense-making process does not only involve finding out what the rules are, but understanding the logic behind the rules. Such sense-making in which people learn that rules are consistent with what they perceive to be acceptable standards of behaviour is likely to increase compliance (Paine 1994).

You have to register as sole trader (self-employed) so that you can claim expenses. Which you want to do—otherwise you’re giving away free tax money! […] When you report income on your tax return, if you’re not registered as self-employed you’d have to put it under a different section and end up paying too much tax.

##### Negotiation

As outlined above, a large number of comments refer to the general rules of paying tax, typically income tax. A different category of comments about laws and rules discusses potential exemptions and ways of using existing rules to pay less tax.

The simplest of cases refers to discussing exemptions (e.g. not having to pay tax for low earnings, etc.).

As for VAT, you wouldn’t have to have a VAT number as long as your taxable earnings are under £68,000 per year. […] When you are asked to provide a VAT number you simply say that you are not VAT registered.

Other discussions refer to the most advantageous ways to save tax, for instance, by making sure all potential work-related expenses are claimed, as illustrated below:

You have to register as sole trader in order to claim expenses. Which is best to do if you’re reporting income—otherwise you’re handing them free tax money!

Or, as shown in the example below, finding the most tax-efficient business structure:

What is the optimal way to sort out my TAX? 1. Invoice to job agency for my contract jobs from my Company […] 2. Register as employee with job agent […] 3. Any other way?

The instances where people attempt to find ways to be more tax-efficient are placed on a continuum from the most common-place practices (e.g. making sure one claims all allowable expenses), to more complicated ways to minimise tax liability, some of which may even be considered tax avoidance, such as a number of comments about how to avoid paying taxes illustrate:

1. I just wondered whether artists need to pay tax on work they sell, if so are there good ways to avoid it (apart from not declaring income), as I know you agree we seem pay tax on pretty much everything. Thanks.
2. Do I still have to pay the tax on work that I’ve picked up from overseas? Thinking if there is any clause whereby I can avoid paying as much tax (hope!).

#### Tax authority

A significant number of comments mention the tax authority. We categorised three ways that taxpayers talk about the tax authority: (1) as a source of expertise about laws and regulations; (2) as an arbiter that people can contact to validate their interpretation of existing rules; and (3) as a subjective law enforcer in relation to which they manage their image as compliant taxpayers.

##### Use expertise

The tax authority is talked about as a source of knowledge about tax laws and regulations; for the most part, people report positive experiences receiving expertise and guidance from the tax authority, and encourage others to seek assistance.

1. […], went to the local tax office and asked them for help. They were fantastically helpful. […] They helped me go through any and all tax liabilities, […]. I mean, it was obvious I wasn’t a tax evader or avoider—I just didn’t have a clue.
2. I’d highly recommend going through the [tax authority] site, or calling if you need help (the sole trader people are very helpful and friendly).

##### Arbitrate

When regulations are unclear or taxpayers feel these can be interpreted in a number of ways, they also relate to the tax authority as an arbiter that can rule whether it regards a certain practice as compliant or noncompliant, such as in the example below:

1. No, seriously, logically thinking it should be fine but the cynicism of the taxman should not be underestimated. I think you should write to them, make your best case, then if they don’t take it as a joke & reply in writing to give you the go ahead you’re covered.
2. Oh, in that case, I would get it confirmed in writing [from the tax authority] if I were you.

##### Manage image

Finally, a number of comments refer to managing one’s image in relation to the tax authority, acknowledging that decisions that a taxpayers is or not compliant are in many cases subjective. There is the concern with what one’s actions may ‘look like’ to the tax authority.

1. Also, consider the sum of the claim versus your total self-employment income or expenses. If it’s small, it’s likely to be more acceptable. [For instance, all my trips are] only 6 % of my total annual expenses. So it doesn’t seem like I’m trying to pull a fast one.
2. Then, when they get back to you I would be honest with them, […]. Be as proactive as possible and you might convince them you’ve goofed and you were not deliberately avoiding them.

#### Tax practitioners

It should be noted that although a significant number of comments mention accountants and tax practitioners, many of these question the need for self-employed individuals to employ the service of an accountant.

1. My first year as a freelancer just ended and I made no profit. […] Do I need an accountant or will I be able to fill in the online form without one?
2. No, you don’t need an accountant. You’ve got it right—sum up the total sales, subtract the expenses and then the result is the profit for taxing.

This second comment reflects the fragility of advice in this arena; it is not actually correct. Of those that stress the need for an accountant, however, the primary relationship with the taxpayer is to *provide guidance*, both in understanding the law and in finding tax savings.

1. BUT it is not legal accounting advice. If in doubt, get some proper advice. Even a small local accountant might give you a consultation cheaply.
2. Your accountant will advise you if you have to register for VAT, so you don’t need to worry about that. This is assuming that your accountant does his job properly.

#### Social network

Finally, a significant number of comments refer to communication and interaction within the taxpayer’s social network that is relevant to tax compliance. Taxpayers share information specific to their network, share own experiences and advice about how to best deal with taxes, and may also try to influence other taxpayers to be compliant, and generally offer support to those who feel anxious.

##### Legal information

As outlined above, most comments refer to tax rules. However, there is an important aspect of how rules are communicated in specific social groups, such as the occupational group. Many taxpayers want to receive information from those in the same occupation and to follow the practice of the occupation—this denotes a role for the norms in the professional group for tax compliance.

1. I guess I should go and talk to the tax office nearer the time but I wanted to speak to other artists first and see what they do.
2. The other nurses I work with have said that transport and meals are allowable expenses and that they’ve claimed for years successfully and have not been audited.

##### Best practice advice

Through interacting with others, taxpayers do not just learn about tax laws, but receive advice from those more experienced, for example, how to keep track of their expenses, how to find a suitable accountant, and so on.

1. Get an accountant—Switch to part time, or get a night job while you start things up—Don’t assume a good April will mean a good May, things are quite seasonal—Mates rates don’t exist—[etc.]
2. I only keep a simple cash book, and use one page for “Money in” and another page for “Money out”.

##### Persuasion

Social networks do not only serve to transmit information—some people actively try to influence others’ actions, especially when compliance decisions are involved.

1. If you are not paying tax, as you previously said, then it is only a matter of time before they get to you and recover what is owed.
2. He needs to deal with it [sort out his tax affairs]. Once the weight is lifted off his shoulders he will feel much better.

##### Support

Finally, social networks serve as support through the often stressful taxpaying process, as illustrated by the comment below.

However, I really wouldn’t worry that much about it. I am sure that it happens a lot. I don’t know the answer but there are lots of people on here who know about this type of thing so one of them should be able to help.

### Assumptions in the Tax Compliance Literature

We set out with the intention to see whether and how our findings match the broad assumptions researchers often make in the tax compliance literature. As outlined in the introductory section, we are not the first to question these assumptions, but we attempt to provide empirical support based on analysing naturally occurring data. We discuss below our findings in relation to each assumption in turn.

1. *Regarding tax behaviour, people’s chief concern is not to decide whether to comply or not* Although the overwhelming majority of studies looking at tax behaviour have focused on tax compliance and the compliance decision, the main concern of taxpayers in our dataset is to find out *how* to comply, to understand and navigate the complex legal landscape. Their chief concern is to make sure they are compliant, rather than to understand how to evade taxes. A relatively small number of cases do discuss practices that may be considered tax avoidance (see also ‘creative compliance’, McBarnet 2004), the concern being to make sure one is compliant with legislation but significantly minimises his or her liability.
  Regarding all taxpayers as potential evaders has been described as a bias in the tax behaviour literature (Kirchler 2007). The focus on tax compliance decisions is also reflected by the few studies that look at tax communication, which propose that taxpayers who talk about tax exchange information about ways to evade tax and avoid detection (Stalans et al. 1991), about the frequency of audits and other people’s evasion behaviour (Hashimzade et al. 2013), and who has been audited in one’s social network (Rincke and Traxler 2009), all of which are topics that feed directly into compliance decisions. While we accept that taxpayers may communicate about these topics, our dataset suggests that their overwhelming concern is to make sense of the rules of tax compliance in order to make sure they are compliant, rather than request information that will help them evade without detection or penalty. Our dataset suggests that the acquisition of tax knowledge is more central than the compliance decision to the tax behaviour of the majority of taxpayers—a majority who are motivated to be compliant and not concerned with ways to evade taxes. Our findings suggest that the majority of taxpayers are compliant; this conclusion echoes wider debates regarding business ethics. An increasing number of authors suggest that the ethical behaviour of business leaders cannot be fully understood through the lens of a rational actor maximising her or his own utility—this leads to an underestimation of ethical behaviour. Instead, many business leaders are driven by ethical principles and values (e.g. Bazerman and Tenbrunsel 2011; Gentile 2010; Messick and Bazerman 1996), although they may possess or report an inflated sense of their own ethics (Randall and Fernandes 1991).
2. *Compliance is not a binary variable* In relation to the compliance decision, the tax compliance literature has widely regarded tax compliance as a binary variable with two potential outcomes: compliant and noncompliant (Kirchler and Wahl 2010; McBarnet, 2004). Considering compliance as a binary decision has important advantages for its measurement; however, compliance behaviour is in reality much more complex than a decision between evasion and full compliance (Braithwaite 2009). As discussed above, compliance in our dataset takes a number of forms, including finding ways to drastically minimise tax liability while complying with the law. In this respect, our dataset echoes other authors in that compliance and noncompliance can take many qualitatively different forms and that studying taxpaying decisions as a binary choice between compliance and evasion does not reflect the complex reality of behaviour (e.g. Braithwaite 2009; Kirchler and Wahl 2010).
  Not only does compliance take many forms, tax evasion is also much less clear in reality than the way it is measured in tax experiments. Since evasion relates to a person’s conscious intention to evade taxes, this personal intention is difficult to assess and distinguish from simple calculation error. As such, assigning to a taxpayer who has filled in a tax return the intention to evade may constitute a subjective decision of tax inspectors (Boll 2013). This concern with the subjectivity of tax evasion judgements is apparent in the online discussions, where people comment on how to make sure honest mistakes are not interpreted as intentional evasion by tax inspectors.
  It becomes apparent that research looking at compliance as a binary outcome (full compliance versus evasion) may lack relevance for the way that compliance plays out in reality, in its many forms shaped by the interaction of taxpayers, tax practitioners, and the tax authority. We hope that future research addresses this gap by looking at the many forms of compliance, including tax avoidance, and also looking at distinguishing between honest error and evasion intention.
3. *The taxpayer and the tax authority are not the only main actors* As Pickhardt and Prinz (2013) argue in their review of tax compliance literature, the main interaction studied in the literature concerns the private relationship between the taxpayer and the tax authority; however, other actors may be as important or even more important for understanding tax behaviour, such as tax practitioners or the taxpayer’s social network. Indeed, more comments in our dataset referred to communication with people in one’s professional group or friends and family, and the involvement of tax practitioners, than comments that referred to the tax authority. Although there has been some interest in the role of tax practitioners (Gracia and Oats 2012; Hite and Hasseldine 2003; Roberts 1998; Stephenson 2010) and taxpayer social networks (e.g. Beers et al. 2013; Hashimzade et al. 2013; Ashby and Webley 2008), more research is needed to understand the role of practitioners and of other taxpayers in taxpayer behaviour.
4. *Taxpayers are seldom concerned with audit rates and penalty levels* Given the classic model of tax compliance decisions based on appraisals of audit probability and penalty levels (Allingham and Sandmo 1972), we would expect high concern in the online discussions with these variables. While audits and penalties may be of interest to taxpayers, they are usually unknown; tax authorities do not usually disclose the number of audits carried out, and while penalty levels are public, they are unknown to many taxpayers (Barham and Fox 2011). As such, in order to estimate the likelihood of audits, people may seek information about recent audits in their social network (Hashimzade et al. 2013). If penalty levels and audit likelihoods were essential to tax behaviour, we would expect people to also seek information online from peers about audits and penalties. However, comments that mention audits are relatively few; of these, for the most part, audits are mentioned as a general deterrent, with no reference to likelihood (see Table 1).
  The largest risk if you don't declare fully is that the tax office will calculate your liability based on similar businesses and issue you with the bill plus fines.
  No comments mention people discussing having been audited or others’ experience of audits. Only a very small number are concerned with the cost-benefit analysis of evasion:
  It depends on the risk you want to take! In my opinion, it’s not worth the risk if your earnings are small.
  It is interesting to observe the term ‘risk’ used in this context. While there is much debate about tax risk management in the context of large business taxpayers (see, for example, Lavermicocca 2011; Mulligan and Oats 2009), there is no research dealing with this aspect of individual compliance. Importantly, although some people mention penalties in general as deterrent, only one instance discusses the actual penalty level, and this is a late-filing penalty.
5. *Alternatively, some taxpayers base their decisions on social norms* An alternative to the view that people comply due to existing deterrents is that people comply due to existing norms against evasion, being motivated to conform to what other people like themselves do (Onu and Oats 2015a; Wenzel 2004). People may be influenced by a multitude of norms, such as national norms, family norms, workplace norms, etc. Since we sourced data from discussion forums for particular professions, the most relevant norms are those in the professional groups (e.g. norms in the hairdressing industry, norms in the IT industry, etc.). A number of comments in the dataset refer to the professional group as relevant for tax behaviour, in particular as a source of specialised knowledge about practice in the profession (Ashby et al. 2009). There is very little indication in our dataset that people discuss norms in terms of approval/disapproval of evasion (i.e. injunctive norms, Cialdini and Trost 1998), but only norms understood as current practice in the profession (i.e. descriptive norms).
  Any artists who know the answer? How do you enter equipment in your accounts?
  Although we cannot tell from analysing the comments whether discussions about current norms in the profession influence behaviour, these types of norms are most likely to influence people when tax rules are ambiguous (Cialdini and Trost 1998). The influence of social norms in the profession on compliance relates more widely to organisational ethical behaviour, where compliance with organisational rules of conduct and the law is chiefly influenced by the existence and promotion of an ethical culture in organisations (National Business Ethics Survey 2013; see also Parker 2000).

## Conclusion

Our study introduces the analysis of naturally occurring online discussions to the study of tax behaviour. We set out to explore discussions about tax among freelancers and small business owners. We found that the ‘universe’ of tax behaviour as reflected in these online discussions is dominated by the need to acquire knowledge about tax laws and regulations, knowledge that will help taxpayers be compliant, but that may also allow them to organise their tax affairs in the most efficient way. Several actors play an important role—the tax authority, tax practitioners, and the wider social network of the taxpayer.

Our analysis highlights several ways in which taxpayers relate to their tax obligations. First, taxpayers seek to make sense of existing regulations. In this sense-making process, the tax authority, tax practitioners, and the wider social networks all play an important role. Second, and perhaps more interesting, taxpayers seek to negotiate compliance boundaries. Many taxpayers seek to understand the various ways of being compliant and choose that which is most efficient for their business. In this process, they rely on their social network to seek out all the alternatives available to them and they rely on the tax authority to arbitrate whether their arrangements are in line with regulations. They also seek out existing norms and practices in their relevant groups (e.g. profession) when deciding how to handle their tax affairs. Taxpayers do not only negotiate being compliant by the way they organise their tax affairs but also by managing their image as honest taxpayers in relation to the tax authority. A small number of the taxpayers in our dataset display noncompliant stances and seek approval in their social network. Further to helping taxpayers understand and negotiate tax regulations, the wider social network also performs other functions: providing general best practice advice in relation to dealing with tax obligations, persuading defiant taxpayers to become compliant, and general social support.

Some of the processes highlighted above have been addressed by past research, such as the acquisition of tax knowledge (e.g. Alm et al. 2010) or the role of social norms in tax behaviour (e.g. Bobek et al. 2007; Wenzel 2004). However, other processes have received less attention. For instance, the fact that taxpayers discuss ways to appear honest so that they are not considered noncompliant highlights the way that compliance is negotiated in the relationship between the taxpayers and the tax inspector (see also Boll 2013). In addition, compliance is influenced by the social network, as taxpayers seek to influence and persuade other taxpayers to comply with regulations (see also Onu and Oats 2015b). The analysis also stresses the various ways in which taxpayers negotiate compliance based on information received from practitioners, tax authority, and the wider social network. Compliance is not clear-cut and a priori defined; it is negotiated in an environment of existing norms and practices (see also Gracia and Oats 2012). This conclusion is not only relevant to tax compliance, but highlights more broadly that ethical business behaviour is not only guided by abstract regulations, but it is also socially situated and negotiated between various actors (e.g. regulators, advisors, wider professional, and social network) (Donaldson and Dunfee 1994).

In addition to analysing the ‘universe’ of taxpayers’ concerns as they appear in spontaneous discussions, we set out to compare these findings to the focus of the tax behaviour literature. Our primary finding is that most taxpayers seem to be unconcerned with the compliance decision; they are motivated to comply and their main concern is to seek information about how to be compliant. However, the focus of the literature on tax behaviour has overwhelmingly been on tax compliance decisions (for a review, see Kirchler 2007). We do not aim to question the validity of research into tax compliance decisions, but rather to question whether taking a compliance decision is relevant to most taxpayers. When compliance is actually discussed, it appears far from a straight-forward binary decision between full compliance and evasion. People may be motivated to be compliant but may misunderstand their obligations or make errors; although they may have not had the intention to evade, they discuss the subjective nature of judgements made by tax inspectors regarding their intentions. People also discuss ways to minimise their tax liability, including practices that could be considered tax avoidance. Our analysis of comments not only suggests that most taxpayers are concerned with being compliant, but that there are many qualitatively different forms of compliance. For the most part, the tax compliance literature has focused on the dichotomy between compliance and evasion. Our analysis, however, suggests that this might be overly reductionist if we are to explain compliance in ‘the real world’, and supports calls for understanding the multiple types of compliance (see also Boll 2013; Braithwaite 2009; Kirchler and Wahl 2010). An understanding of tax behaviour beyond the dichotomy of compliance/noncompliance should be of particular interest to policy makers who seek to understand practices such as ‘aggressive tax planning’ or ‘tax avoidance’ as they are negotiated in practice (see Gracia and Oats 2012).

We also looked at whether people are concerned with finding out about audits and penalties, or social norms regarding evasion, as suggested by the compliance literature (see Allingham and Sandmo 1972; and Wenzel 2004, respectively). We found little evidence of people being concerned with finding out about audit probabilities or penalty levels. There was also little evidence of people discussing their approval or disapproval (i.e. injunctive norm) of tax evasion. However, some people did discuss what others do (descriptive norm, Bobek et al. 2007) in relation to their tax affairs and taxpayers are even actively encouraged to comply (see also Onu and Oats 2015b). These findings are indicative of the fact that the wider social environment can have a positive effect on business ethical decisions (Christie et al. 2003). It is important to note that our conclusion does not aim to question the validity of previous research on the effect of deterrence factors on tax behaviour on those people who do indeed consider evading, but to question whether many people consider evasion at all. Our aim is thus to question the focus of research on tax behaviour on evasion decisions and propose that other topics may be essential to understanding the experience of the majority of compliant taxpayers. Such avenues include further research into people’s acquisition and understanding of complex tax regulations, and the role of taxpayers’ social networks and their tax practitioners in this process. Also, a deeper understanding of the different manifestations of compliance is desirable to focusing on the dichotomy of compliance–evasion.

The focus of research on tax behaviour has important practical implications for informing tax authority activities. A research field focused on the evasion decision will advise tax administrations to focus on audits, publicising large penalties, or ‘naming and shaming’ evaders, and support an ‘enforcement’ paradigm in dealing with taxpayers (Alm et al. 2010). However, the academic focus on deterring evasion will bring little contribution to all those areas of tax administration that affect the large majority of compliant taxpayers, areas such as educating people about their tax obligations, making sure they are confident enough to approach authorities, that they find it easy to comply. Despite the fact that ‘the service approach’ is increasingly at the core of tax administrations around the world, few academic researchers conduct research to inform how to support compliant taxpayers (for an exception, see, for example, Alm et al. 2010). Although our analysis has dealt with the compliance of individuals, similar analyses of online discussions may be helpful to understand attitudes and opinions related to the tax compliance of large taxpayers (see, for example, Christians 2012; Datt 2014).

It is important to note that our study relies on people’s public discussions, and it is likely that people may discuss compliance decisions to a larger extent in private settings. This limitation is common to all other studies of tax compliance that rely on self-report, such as those employing interviews, large-scale surveys, or experiments where compliance behaviour is observed; given that tax evasion is often perceived as immoral and illegal, people are reluctant to admit to noncompliance to peers or to a researcher. Despite this limitation, self-reports are central to tax compliance research and many seminal works in the field rely on self-report measures. And although there is debate regarding the extent to which self-reports reflect people’s behaviour, self-reports are considered to be very useful albeit imperfect proxies for actual compliance behaviour (for a discussion, see Elffers et al. 1992). Although our study employs self-report, we use naturally occurring discussion among peers, which often provide more accurate reflections of actual behaviour than self-reports given in interviews or surveys because they avoid the researcher biasing responses (Wetherell et al. 2001; Wooffitt 2005).

It is also important to note that we do not aim to provide conclusions that are representative of all taxpayers. Our dataset is limited to self-employed professionals and owners of very small businesses, who carry out their business in the UK and who are accustomed to interacting in an online environment. Nonetheless, it is likely that the content of communications about tax between these professionals is similar to those like them operating in other countries, or discussing tax in other types of public fora. People who run more established businesses or those with particular knowledge of tax may display different behaviour. The forums selected in our dataset are large and reputable online communities. It may be that in more private settings (such as face-to-face communication or online communication in smaller private groups), people may be more likely to discuss noncompliance decisions rather than seek information about how to comply, but having no access to such private discussions it is difficult to assess to what extent this is true. Overall, despite the limitations inherent in analysing public self-report data, we believe that the current study is valuable in offering an in-depth exploration of naturally occurring discussions.

We hope other researchers will seek to analyse naturally occurring data to investigate tax behaviour and business ethical decisions more generally. The online environment provides vast opportunities for collecting such data through the use of social media sites, forums, news websites and user comments sections, blogs, etc. (for overviews, see Hine 2013; Kozinets 2015). Equally, more traditional sources can be included, such as transcripts of boardroom discussions, media programme transcripts, letters to editors of news outlets, and others. The great advantage of such data is that they are produced with no interference from the researcher, not having been elicited by survey questions or an experimental design, and provide the opportunity to explore the topics that are most important and relevant to the people we study.
